# Structural Identification of Metalloproteomes in Marine Diatoms, an Efficient Algae Model in Toxic Metals Bioremediation

**DOI:** 10.3390/molecules27020378

**Published:** 2022-01-07

**Authors:** Christos T. Chasapis, Massimiliano Peana, Vlasoula Bekiari

**Affiliations:** 1 Department of Animal Production, Fisheries and Aquaculture, University of Patras, 30200 Messolonghi, Greece; 2 Department of Crop Science, University of Patras, 30200 Messolonghi, Greece; bbekiari@upatras.gr; 3 Institute of Chemical Engineering Sciences, Foundation for Research and Technology, Hellas (FORTH/ICE-HT), 26504 Patras, Greece; 4 Department of Chemistry and Pharmacy, University of Sassari, 07100 Sassari, Italy

**Keywords:** diatoms, marine pollution, metal detoxification, toxic metal, metalloproteome

## Abstract

The biosorption of pollutants using microbial organisms has received growing interest in the last decades. Diatoms, the most dominant group of phytoplankton in oceans, are (i) pollution tolerant species, (ii) excellent biological indicators of water quality, and (iii) efficient models in assimilation and detoxification of toxic metal ions. Published research articles connecting proteomics with the capacity of diatoms for toxic metal removal are very limited. In this work, we employed a structural based systematic approach to predict and analyze the metalloproteome of six species of marine diatoms: *Thalassiosira pseudonana*, *Phaeodactylum tricornutum*, *Fragilariopsis cylindrus*, *Thalassiosira oceanica*, *Fistulifera solaris*, and *Pseudo-nitzschia multistriata*. The results indicate that the metalloproteome constitutes a significant proportion (~13%) of the total diatom proteome for all species investigated, and the proteins binding non-essential metals (Cd, Hg, Pb, Cr, As, and Ba) are significantly more than those identified for essential metals (Zn, Cu, Fe, Ca, Mg, Mn, Co, and Ni). These findings are most likely related to the well-known toxic metal tolerance of diatoms. In this study, metalloproteomes that may be involved in metabolic processes and in the mechanisms of bioaccumulation and detoxification of toxic metals of diatoms after exposure to toxic metals were identified and described.

## 1. Introduction

Anthropogenic activity mainly due to mining, technological activities, and agricultural applications have led to a vast dispersion of toxic metals in natural environments [1,2,3]. Toxic metals are introduced into the atmosphere, soil, coastal, and marine environments through a variety of sources, including emissions and wastewater from metal-based industries, improper waste disposal, as well as from household effluents [4,5]. Once dispersed, toxic metals can be taken in by humans through food, water, and air and accumulated in the body, affecting various biological functions and causing multiple organ damage and serious diseases such as cancer [1,6]. Thus, environmental pollution of toxic metals (in the form of their ions) is a problem of great concern, leading to increasing interest in the scientific community in developing methodologies to address and reduce their harmful effects.

In recent years, techniques that utilize biological mechanisms of microorganisms and plants to eradicate hazardous contaminants and restore polluted environments to their original condition are increasingly being used. Thanks to their characteristic enzymes and biological processes such as bioaccumulation, biomineralization, bioabsorption, and biotransformation, these organisms maintain the homeostasis of toxic metals and use them as a source of energy for their growth and development [7,8]. In this way, diatoms have developed resistance to toxic metals, adapting their metabolism in order to survive in an environment polluted with these contaminants [8,9,10].

Diatoms are the most dominant and diverse group of phytoplankton, which account for 45% oceanic primary productivity due to their higher growth rate and competitive characteristics over other groups of microalgae [11]. Diatoms are unicellular eukaryotic microscopic plants with approximately 200 genera and more than 100,000 species. Diatom cells exist within a unique silica cell wall, known as frustules, which is synthesized intracellularly by the polymerization of silicic acid monomers. During biosorption and bioaccumulation (when algae adsorb toxic metals), exposed parts of the diatoms undergo substitution of different metal ions or complexation with toxic metals at the frustules [12]. Furthermore, the principal organic constituents of a diatom’s cell walls, peptidoglycans, polysaccharides, lipids, and proteins, act as biotic ligands for metal binding [13,14]. Molecular mechanisms of diatoms enables them to differentiate between essential and non-essential metals (for human life) for their growth and development [10,15,16]. The metal binding to the surface depends on metal requirements for intracellular metabolic activities and consequently some toxic metal ions are transported into the cells and utilized for different metabolic functions [10]. Upon their accumulation, the microalgae produce reactive oxygen species (ROS), which act as signaling molecules in order to control cell metabolism [17] and induce a programmed cell death process in various diatom species [18,19].

Diatoms have attracted considerable attention due to their success in various environmental conditions, and studies that utilize genetic manipulation and metabolic engineering are needed to provide insight into their adaptation capacity [20]. Due to their short life cycles, diatoms are used as bioindicators of water quality in rivers, lakes, and oceans by changing their diversity and density [21,22,23], and recently diatom applications for specific industrial wastewater treatment have been investigated [24].

Though diatom algae are one of the most studied species in terms of cellular and molecular responses to metal toxicity, the identification of metalloproteome that is involved in their characteristic biosorption machinery is very limited. The holistic identification of the metallo-binding proteins/enzymes and the elucidation of possible correlations with the metabolic pathways responsible for metal sequestration by diatoms is a crucial prerequisite for their molecular manipulation in order to improve survival rates and stability when exposed to high metal concentration and to discover novel artificial bio-chelators for toxic metals decontamination [20,25].

In this work, we identified the metalloproteomes for essential (Zn, Cu, Ca, Co, Fe, Mg, Mn, Ni) and non-essential (Cd, Hg, Pb, Ba, Cr, As) metal ions for six marine diatoms species: *Fistulifera solaris*, *Fragilariopsis cylindrus*, *Phaeodactylum tricornutum*, *Thalassiosira pseudonana*, *Thalassiosira oceanica*, and *Pseudo-nitzschia multistriata*, by applying a systematic structure-based approach. This study allowed highlighting possible molecular linkages between the metalloproteome of diatoms and their cellular mechanism of bioaccumulation, homeostasis, and detoxification after their exposure to an environment contaminated by toxic metals. Their intracellular metabolic activities and their unique adaptation in marine environments poor in essential metals are also discussed.

## 2. Materials and Methods

### 2.1. Identification of Diatom Metalloproteomes

Metal-binding proteomes of six marine diatoms species (Table 1) have been identified by applying a systematic structure-based approach used previously to propose putative metalloproteomes in various organisms, such as bacteria, viruses, and toxicological models [26,27,28]. The approach combines strategies based on structural data and annotation to identify known metal-binding domains in their sequences [29,30].

All 3D structures of Zn^2+^ Cu^1+^/^2+^, Ca^2+^, Co^2+^/^3+^, Fe^2+^/^3+^, Mg^2+^, Mn^2+^/^4+^, Ni^2+^, Cd^2+^, Hg^2+^, Pb^2+^, Ba^2+^, Cr^2+/3+^ and As^3+^—binding proteins have been provided by the Protein Data Bank (PDB) (https://www.rcsb.org/, accessed on 10 November 2021) and MetalPDB (https://metalpdb.cerm.unifi.it/, accessed on 10 November 2021) [31]. From these structures, each specific metal-binding pattern (MBP) involved in the interaction of specific proteins with the specific metal was extracted. This set served as the starting point for the search of MBP in the diatom proteomes. All the proteomes were downloaded from the protein resource UniProt (https://www.uniprot.org/, accessed on 10 November 2021) [32]. Then, to achieve maximal identification coverage, all the diatom proteomes were analyzed for the relevant Pfam (protein families) metal-binding domains [33]. Each protein was scanned for the occurrence of Pfam domains using the HMM search function of HMMER 3.1b2 (http://hmmer.org/, accessed on 10 November 2021) [34] and a threshold of 0.05 for the E value (as calculated by Pfam). False positives were filtered out by searching MBPs in the retrieved protein sequences, and by rejecting those lacking the MBPs. The MBP filter was applied, assuming that the predicted proteins contained all the ligands of the MBP with spacing within each sequence maintained within ±20% of total number of amino acids (or ±1 amino acid for short spacing).

### 2.2. Functional Classification, Gene Ontology Annotation, and Localization of Metal-Binding Proteins

Functional classification of metalloproteins was obtained using DAVID web server (https://david.ncifcrf.gov/, accessed on 10 November 2021) [35]. Gene Ontology (GO) terms were retrieved by ClueGO plug-in [36] of Cytoscape v.3.0.0 [37] and the KEGG pathway annotation [38]. The subcellular localization of metalloproteins was based on UniProt web source [32].

## 3. Results

### 3.1. Non-Essential Metal Binding (Non-EMB) Proteome of Six Diatom Species

**Figure 1:**
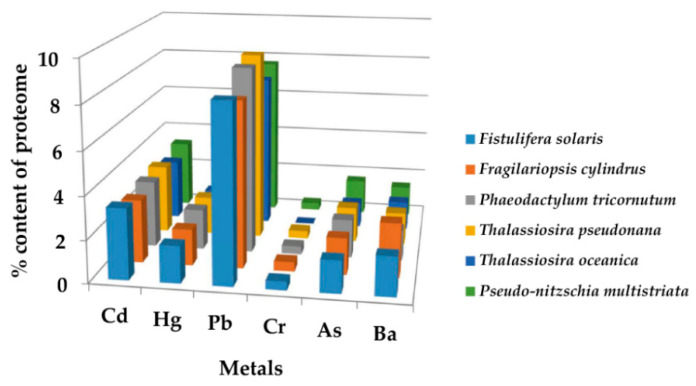
The percentage content of non-EMB proteins in the proteomes of the six diatoms analyzed.

**Figure 2:**
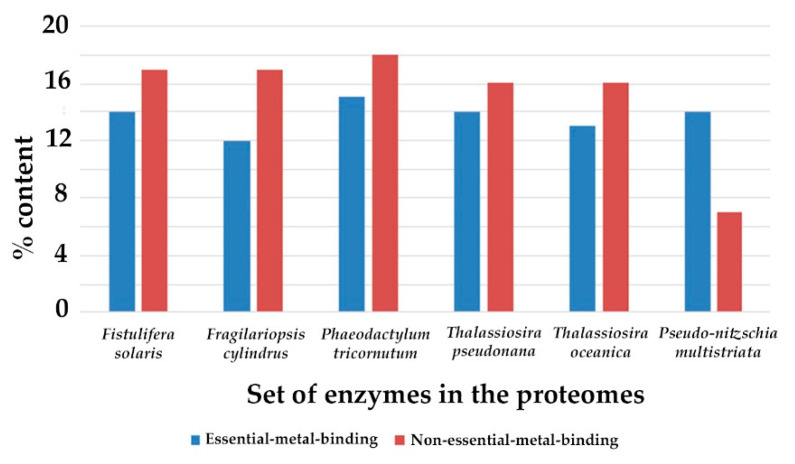
The percentage of metal binding enzymes in the total number of enzymes for the six diatom species analyzed.

The identified metalloproteins bound to non-essential metals (relative to the sum of Cd, Hg, Pb, Ba, Cr and As proteins), represent 11%, 10.5%, 8.3%, 8.2%, 8%, and 8% of the *Thalassiosira pseudonana*, *Phaeodactylum tricornutum*, *Pseudo-nitzschia multistriata*, *Fistulifera solaris*, *Thalassiosira oceanica*, and *Fragilariopsis cylindrus* proteome, respectively. All the non-essential metal binding (non-EMB) proteins (for the six diatom species) are listed in the Supplementary Materials (datasheet-1) and their percentage contents per species are illustrated in Figure 1. Recurrent proteins that bind to different metals were identified and the average percentage of unique binding proteins for the six diatoms per metal type was calculated: 2.2%, 0.01%, 6.8%, 0.01%, and 1.2% for Cd, Hg, Pb, Ba, Cr, and As, respectively. The Pb-binding proteins showed, in all the diatom proteomes, the highest content (up to 9%), followed by Cd (up to 3%), Hg, As and Ba (up to 1.9%), and Cr (less than 1%). The majority of non-EMB proteins is localized in the membrane (62%), then in the cytoplasm (12%), and the ribosome (12%). The average enzymatic content in the non-EMB proteomes of six diatom species is ~53%. The percentage of non-EMB binding enzymes in the total number of enzymes for each diatom species and their classification, based on Enzyme Commission (EC) numbers, are presented in Figure 2 and Table 2.

The most significant molecular function GO terms represented in non-EMB proteomes refer to catalytic activity (53%), transmembrane transporter activity (11%), structural constituent of ribosome (7%), and antioxidant activity (1%). The majority of the proteins that are ribosomal and involved in antioxidant activity are Pb- and Hg-binding molecules, respectively. Sixty-four percent of the enzymes are involved in metabolic processes, taking part in a complex sequence of controlled biochemical reactions (metabolic pathways) which allow diatoms to grow, maintain, and respond to environmental changes. In particular, the majority of them are involved in the production of primary metabolites lactic acid and certain amino acids (87%) and in nitrogen metabolism (87%). The rest are involved in catabolic pathways: break down of carbon compounds with release of energy used by diatom cells (17%) and in methylation (6.7%).

### 3.2. Essential Metal Binding (EMB) Proteome of Six Diatom Species

**Figure 3:**
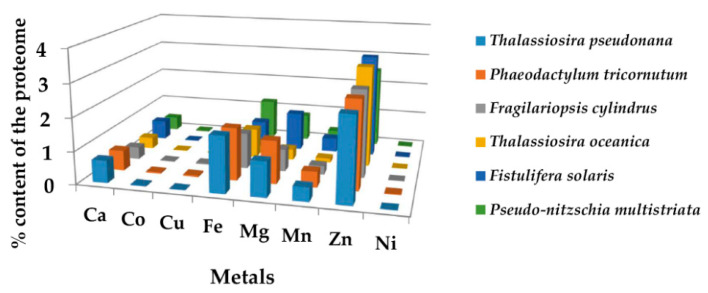
The percentage content of EMB proteins in the proteomes of the six diatoms studied in the present work.

The identified EMB proteins (relative to the sum of Zn, Cu, Ca, Co, Fe, Mg, Mn, and Ni proteins) represent 6.2%, 5.9%, 5.6%, 4.9%, 4.7%, and 4.1% of the *Fistulifera solaris*, *Phaeodactylum tricornutum*, *Thalassiosira pseudonana*, *Pseudo-nitzschia multistriata*, *Fragilariopsis cylindrus*, and *Thalassiosira oceanica* proteome, respectively. All the EMB proteins are listed in the Supplementary Materials (datasheet-2) and their percentage contents per species are illustrated in Figure 3. Recurrent proteins that bind to different metals were identified and the average percentage of unique binding proteins for the six diatoms per metal type was calculated: 2.9%, 0.08%, 0.5%, 0.0%, 1.3%, 0.9%, 0.5%, and 0.09% for Zn, Cu, Ca, Co, Fe, Mg, Mn, and As, respectively.

In all species of diatoms, the Zn-binding proteins have the highest content (up to 3%), followed by Fe (up to 1.5%), Mg (up to 1%), Mn and Ca (up to 0.5%), and Co, Cu and Ni (less than 0.1%). The majority of EMB proteins is localized in the membrane (55%) and then in the cytoplasm (11%). The average enzymatic content in the EMB proteomes of the six diatom species is ~33%. The percentage of EMB binding enzymes in the total number of enzymes for each diatom species and their classification, based on Enzyme Commission (EC) numbers, are presented in Figure 2 and Table 2. The most significant molecular function GO terms represented in EMB proteomes refer to catalytic activity (33%), molecular function regulator (2%), antioxidant activity (1.7%) and transcription regulator activity and transmembrane transporter activity (less than 1%). Eighty-four percent of the identified enzymes are involved in metabolic pathways. The majority of them is involved in the production of primary metabolites (73%), in nitrogen metabolism (66%), in catabolic pathways (11%), and in methylation and protein glycosylation (3% and 1.4%, respectively).

### 3.3. Non-Essential Metal Binding in Essential Metal Binding Sites in Diatom Metalloproteins

**Figure 4:**
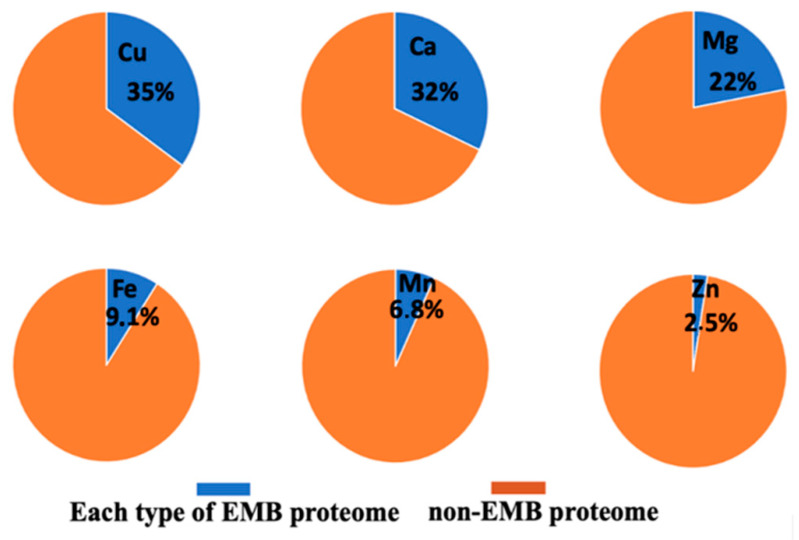
Average protein overlaps between EMB-proteome (for Cu, Ca, Mg, Fe, Mn, and Zn) and non-EMB proteome for the six diatom species studied.

Since diatoms are microorganisms with a unique toxic metal tolerance and a mechanism that utilizes toxic metals in the catalytic site of enzymes in the absence of the associated essential metal, in this work, we attempted to discover possible EMB proteins that could bind a toxic metal. Based on the present analysis, the content of the proteins with EMB motifs that was identified in the non-EMB proteome in all six diatom proteomes ranges from 6 to 7%. Specifically, the averages of the possible binding rates of non-essential metals to each type of EMB motif in diatom proteomes are presented in pie charts in Figure 4.

Based in the present analysis, the most important diatom enzymes that share EMB and non-EMB motifs are: (i) Cu^2+^ transmembrane transporter and electron transfer activity (for instance plastocyanin), (ii) Ca^2+^ binding proteins (including Calmodulin, Phosphoinositide phospholipase C and Peptidylprolyl isomerase), and (iii) Mg^2+^ transmembrane transporters and Mg^2+^ binding proteins with catalytic activity, including transferring phosphorus-containing groups, protein kinase, and hydrolase activity.

## 4. Discussion

This work describes, for the first time, an in silico identification of the metalloproteome for marine diatoms, a group of phytoplankton efficient in assimilation and detoxification of toxic metal ions. The results indicate that the metalloproteome constitutes a significant proportion of the total proteome of diatoms (~13%). Specifically, in the six species of diatoms analyzed, the non-EMB proteome is significant larger than those observed for essential metals, (for instance in *Thalassiosira oceanica*, they are 8% and 4.1%, respectively). In EMB proteome, the Zn-binding proteins have the highest content (up to 3%), followed by Fe (up to 1.5%), Mg (up to 1%), Mn and Ca (up to 0.5%), and Co, Cu and Ni (less than 0.1%). These results are in agreement with data from other organisms: Zn-binding proteins have the highest content and constitute up to 9%, 5%, and 6% of the metalloproteome in Eukarya, Bacteria, and Archea, respectively [39]. In eukaryotes and prokaryotes, the size of the Cu proteome was below 1% [40]. The higher frequency for the Zn-binding proteins was somehow expected because Zn^2+^ is one of the most abundant metal ions in living organisms and it is bound to proteins or enzymes which are involved in a variety of fundamental biological processes.

In non-EMB proteome, the Pb-binding proteins showed the highest content (up to 9%), followed by Cd (up to 3%), Hg, As and Ba (up to 1.9%), and Cr (less than 1%). Τhe above non-EMB protein contents are comparable to those previously reported for *Tetrahymena* sp. [26,41]. *Tetrahymena* are ciliated protozoa that inhabit streams, lakes, and ponds and are mostly known for their use as a tool for toxicological studies and “whole-cell biosensor” (WCB) for detecting toxic metals pollution in aquatic or soil samples [26,28]. Interestingly, in diatoms, the content of enzymes is higher in non-EMB compared to EMB proteome. That means that toxic metals may be involved in large-scale metabolic processes and multiple cellular pathways. In fact, the majority of non-EMB proteins were enzymes involved in organic substances, nitrogen compounds, biosynthetic and primary diatoms metabolic processes, and the rest of them has important biological functions (including antioxidant activity and transmembrane transporter activity).

Various toxic metals ions, including Cd^2+^ may bind to Zn^2+^ and Cu^2+^-coordination motifs of proteins in archaeal, bacterial, and eukaryotic organisms (metallothioneins [42], zinc transporters [26,43] and proteolytic enzymes [44], RING domains of E3 ligases [45,46], superoxide dismutase [47,48], catalase [49,50], and glutathione reductase [51,52]). Also, previous studies have shown that ion sensors like calmodulin (CaM, calcium sensor) are activated effectively by other metals (i.e., Pb^2+^ and Ba^2+^), in fact with higher affinity than Ca^2+^ [53,54,55]. These observations could be explained, at least in part, by a similar coordination geometry for both Ca^2+^, Pb^2+^ and Ba^2+^ in the EF-hands of CaM, suggesting similar mechanisms when CaM is activated by different metal ions. It was proposed that the toxicity-related activation mechanism of CaM by lead may have two aspects: hyperactivation at low concentration of Pb^2+^ and inactivation at high concentrations, leading to the possible study of this molecule as a concentration sensitive sensor for Pb^2+^ [53]. According to the present study, predicted non-EMB proteins are enzymes with typical Zn-, Cu-, Ca-, Co-, Fe-, Mg-, Mn-, and Ni-binding motifs. This observation is in agreement with the remarkable ability of diatoms for the biogeochemical cycling of cadmium and their significant competitive advantage to survive in an oceanic environment poor in essential metals, but rich in toxic metals (usually Cd). Diatoms utilize Cd^2+^ as a catalytic metal ion in carbonic anhydrase, leading to lowest cadmium concentrations in surface water than the depths [56,57].

Our study reveals that essential metal binding motifs that could utilize non-essential metal ions belong mostly to proteins with metal ion transporter activity: Copper transporter, CaM (Ca^+2^-transporter), H(+)-exporting diphosphatase (Mg^+2^-transporter), and Transmembrane protein 163 and PHD domain-containing protein (Zn^+2^-transporters). These results suggest that Ca, Mg, Cu, and Zn proteins and enzymes could be involved in toxic metal homeostasis, subcellular distribution, and detoxification, after diatom’s exposure to toxic metals, since essential metal transmembrane transporters may constitute the main candidates for non-essential toxic metal uptake in diatoms.

Diatoms are excellent biological indicators, and they are used as bio-monitors of pollution. Several studies indicate that diatoms are pollutant-resistant species which varies from one species to another in response to their habitat and the types of metals they are exposed to [10]. This study reveals the order Pb^2+^ > Cd^2+^ > Hg^2+^ for the toxic metal binding capability of diatom proteomes. According to the literature, *Thalassiosira pseudonana* and *Phaeodactylum tricornutum*, have been characterized as toxic metal tolerant diatom species and potential candidates for toxic metal removal applications [58,59]. Since these two diatoms have a slightly higher content of non-EMB proteins compared to the others, we can suppose that the other four diatoms (*Fragilariopsis cylindrus*, *Thalassiosira oceanica*, *Fistulifera solaris*, *Pseudo-nitzschia multistriata*) probably exhibit the same toxic metal tolerant capacity.

The volume of research work done on application of diatoms for toxic metal removal is quite sparse compared to green and blue green algae [12]. Some reports revealed possible biosorption and bioaccumulation of toxic metals by diatoms. For instance: (i) in *Thalassiosira oceanica*, the Cd absorption process involves Cd intake during Fe deficiency [60,61], (ii) in *Thalassiosira pseudonana*, Cd, Cu, and Zn absorption involves antioxidant activity, ROS scavenging [62], and (iii) in *Phaeodactylum tricornutum*, Cd, Pb, and Cu absorption involves phytochelatins and antioxidant enzymes [15,63,64]. Based on our results, the proteins which are involved in the experimentally reported Cd absorption of *Thalassiosira oceanica* are proteins with Cd-binding motif and cation transporter activity such as: the ATPase-coupled cation transmembrane transporter (HMA domain-containing protein, UniProt ID: K0SDM0), and the ion trans domain-containing protein (UniProt ID: K0S7Z5), and intracellular calcium binding receptor CaM (UniProt ID: K0T731). In *Thalassiosira pseudonana*, the reported Cd, Cu, and Zn absorption may be facilitated through proteins with binding motifs that could replace essential metals (Cu, Zn, and Fe) by non-essential ones (Cd) and antioxidant activity such as: Superoxide dismutase (UniProt ID: B8C2J5), Cytochrome C peroxidase (UniProt ID: B5YMA2), Ascorbate peroxidase (UniProt ID: B8CFA9), and Catalase-peroxidase (UniProt ID: B8CF21). Finally, in *Phaeodactylum tricornutum*, the mechanism of the reported absorption of toxic Cd and Pb may involve antioxidant enzymes with binding motifs that could substitute Fe by Cd and Pb, such as Catalase-peroxidase (UniProt ID: B5Y4Y9), Ascorbate peroxidase (UniProt ID: B7G384), and Superoxide dismutase (UniProt ID: B7FPQ3).

## 5. Perspectives

In the present work, metalloproteome of six species of marine diatoms: *Thalassiosira pseudonana*, *Phaeodactylum tricornutum*, *Fragilariopsis cylindrus*, *Thalassiosira oceanica*, *Fistulifera solaris*, and *Pseudo-nitzschia multistriata* was analyzed. The main outcomes are the discovery of possible molecular linkages between metalloproteome of diatoms and (i) their cellular machinery for the toxic metal bioaccumulation, homeostasis, and detoxification after diatom’s exposure to toxic metals, (ii) their intracellular metabolic activities, and (iii) the unique adaptation of diatoms to marine life in essential metal-poor environments. As the published research on proteome level regarding the application of diatoms for toxic metal removal is limited, this work and the provided list of diatom metalloproteins could be useful for future studies related to individual metal binding enzymes of various diatoms and offer insights useful for metabolic engineering efforts for biotechnological production of strains with high-toxic metal removal performing. Moreover, these results are of interest in ecotoxicology, where the mechanisms underlying the metal tolerance in marine diatoms remain a matter of research. The discovering of novel natural or artificial bio-chelators of toxic metals would broaden the spectrum of their metal binding affinity and consequently the collection of metal contaminants in polluted water by diatoms as transgenic plants are not able to do so without soil. Furthermore, the optimization of metal recovery by diatoms could be even used to recover raw materials from wastewater, reducing the cost of their purification.

## Figures and Tables

**Table 1:** UniProt IDs and proteome size of the six species of diatoms studied.

Proteome ID	Organism	Strain	Number of Proteins	Number of Characterized Proteins in UniProt Database
UP000198406	*Fistulifera solaris*	JPCC DA0580	20.319	10.169
UP000095751	*Fragilariopsis cylindrus*	CCMP1102	18.075	7.692
UP000000759	*Phaeodactylum tricornutum*	CCAP 1055/1	10.465	10.447
UP000001449	*Thalassiosira pseudonana*	CCMP1335	11.718	6.672
UP000266841	*Thalassiosira oceanica*	CCMP1005	34.431	6.681
UP000291116	*Pseudo-nitzschia multistriata*	B856	11.907	4.108

**Table 2:** Classes of the enzymes and their average % content in non-EMB and EMB in proteomes of the six diatom species studied in this work.

Enzyme Class	E.C Number	Average % Content	
		EMB Proteome	Non-EMB Proteome
Oxidoreductases	1	13	6
Transferases	2	24	19
Hydrolases	3	30	27
Lyases	4	6	2.5
Isomerases	5	2.4	2
Ligases	6	5	0
Translocases	7	3.4	0

## Data Availability

Not applicable.

## Supplementary Materials

The following supporting information can be downloaded online. datasheet-1 (non-EMB proteome), datasheet-2 (EMB proteome).

## Sample Availability

Samples of the compounds are not available from the authors.
